# Assessment of Optimal Treatment Strategies and Their Outcomes in T3N1 Rectal Cancers

**DOI:** 10.7759/cureus.73139

**Published:** 2024-11-06

**Authors:** Atreya Subramanian, Almira Dias, Adel Hamed, Gethin Williams, Usman Khan

**Affiliations:** 1 General Surgery, Aneurin Bevan University Health Board, Newport, GBR

**Keywords:** disease free survival (dfs), distant metastases, neoadjuvant, overall survival (os), rectal cancer, recurrence local, surgery

## Abstract

Background*:* This study compares the outcomes of a surgery first vs a neoadjuvant treatment strategy in T3N1M0 rectal cancers.

Methods*:* This was a single-centre retrospective cohort study of patients admitted for curative treatment of T3N1 rectal cancer. Patients with pre-treatment T3N1 and pathological T3N1 disease were included in the study. Patients were divided into two groups depending on whether they had surgery or neoadjuvant therapy as their initial phase of treatment. Primary outcome measures were local recurrence and distant recurrence. Secondary outcomes were disease-free survival (DFS) and overall survival (OS). Tabulated results were analyzed with appropriate statistical tests.

Results*:* One hundred and ten patients were initially selected. Fourty-eight were finally included after excluding patients who did not meet the staging criteria or were not eligible for curative treatment. Twenty-nine patients underwent surgery, and 19 patients with neoadjuvant therapy as their first treatment. No local recurrence was noted in either group, with a distant recurrence noted in group 2 (6.9%) and group 1 (5.26%) cases among the surgery-first and neoadjuvant-first groups, respectively. Disease-free survival and overall survival were 29.5 and 30 months for the surgery-first group and 22 and 22 months for the neo-adjuvant group, respectively.

Conclusions*:* Outcomes in the surgery-first group were non-inferior to that of the neoadjuvant group. A threatened circumferential resection margin (CRM) on pretreatment staging warrants neoadjuvant therapy to ensure an R0 resection. Extramural vascular invasion (EMVI), being a negative prognostic factor, doesn’t preclude a surgery-first approach.

## Introduction

Despite newer advancements in the management of rectal cancer, there are still varied opinions regarding the optimal treatment strategy for patients with T3N1 disease, highlighted by a recent “Gloves off” debate conducted in the British Journal of Surgery in November 2023. Each approach, i.e., surgery first, systemic anticancer therapy (SACT), total neoadjuvant therapy (TNT), and long-course chemoradiotherapy (LCRT), was considered and advocated for by leaders in their respective fields [1-4].

Neo-adjuvant therapy in any form does have its share of side effects. Radiotherapy has been associated with increased bowel frequency, fecal incontinence, radiation enteritis, and secondary malignancies [5,6]. The PROSPECT trial also acknowledges the toxicity secondary to anticancer therapy (excluding RT) in the form of mucositis, diarrhoea, vomiting, and neuropathy [7].

Rectal surgery is also associated with significant morbidity, including poor body image (stoma), gastrointestinal, bladder, and sexual dysfunction [8]. The wait-and-watch strategy is a potential solution in this regard, providing a cure without surgery (when neoadjuvant therapy provides a complete clinical response). The recent population-based “Norwait study” has questioned this strategy as it showed a 53% regrowth rate at a median follow-up of 54 months, higher than most other studies (attributable to mucosal disease in complete clinical response (CCR) cases) [9]. A Memorial Sloan Kettering (MSK) study in non-responders demonstrated that a delay in surgery by a span of eight weeks resulted in worse disease-free and cancer-specific survival rates [10]. This would imply an individual who would have ordinarily had surgery and missed their timely follow-up for some reason would have a worse outcome.

It is clear that the decision-making process is fraught with complexities, and multiple factors need to be considered, including patient preference. This study aims to compare the pathological and oncological outcomes of a surgery-first approach versus a neo-adjuvant approach in patients with T3N1 rectal cancer.

## Materials and methods

This was a single-centre retrospective study servicing a catchment area with a population of approximately 650,000. The study involved patients with primary rectal cancer treated with a curative intent between the 1st of January 2021 and the 31st of May 2023 and was conducted and reported in accordance with STrengthening the Reporting of OBservational studies in Epidemiology (STROBE) methodology for observational studies [11]. 

Data was obtained from a centralized trust database, prospectively maintained for the National Bowel Cancer Audit (NBOCA). This included data from radiology, pathology, and oncology records (including operative notes). All data was saved/accessed on a secure trust-based server to maintain patient confidentiality. Demographics, preoperative staging, intraoperative findings, histopathological findings, and follow-up data were collected and analyzed. Primary outcome measures were local and distant recurrence. Secondary outcomes were disease-free survival (DFS) and overall survival (OS).

All patients were staged clinically with a pelvic MRI and a CT (abdomen and pelvis), along with a histological diagnosis achieved via colonoscopy/ sigmoidoscopy in accordance with the ESMO guidelines [12]. All patients were discussed in a multi-disciplinary team meeting (MDT) before deciding on treatment. If the intent was deemed to be curative, a decision on the modality was taken (surgery first vs neoadjuvant) based on T3 substage, distance from the anal verge, threatened circumferential resection margin (CRM), and extramural vascular invasion (EMVI) positivity.

All patients with a pre-treatment MRI staging of T3N1 disease were included; subsequently, patients who went straight to surgery and had a final pathological staging of T3N1 disease were also included. Patients who were too frail for curative treatment or found to have metastatic disease on initial clinical staging were excluded.

Short-course radiotherapy (SCRT) (5Gy/fraction x 5 days) was given either alone or in conjunction with four cycles of capecitabine and oxaliplatin (CAPOX, as part of TNT). Patients receiving LCRT had 45Gy over 25 fractions along with 5-fluorouracil (5-FU). This was followed by surgery (anterior resection/abdominoperenial excision of the rectum) [12,13].

Standard operative techniques were employed by six consultant colorectal surgeons. Laparoscopic surgery was performed when feasible. Anterior resection was done with total mesorectal excision. Standard abdominoperineal excision was carried out in the Lloyd Davies position according to the operative principles of Miles' original description, with cylindrical perineal dissection [14].

All patients were followed up for a minimum of 14 months (most for 18 months or more) having at least one post-operative CT scan and regular six monthly carcinoembryonic antigens (CEA) blood level checks. The year 1 colonoscopy, in accordance with the NICE guidelines, was booked for all patients [15].

Definitions: Tumours were defined as low (0-5 cm from the anal verge), mid (5-10 cm), or high (10-15 cm) rectal based on preoperative endoscopic/on-table rigid sigmoidoscopic findings, and according to where the majority of the tumour was located. Threatened CRM involvement (pre-operative) was defined by the presence of tumour foci (primary, nodal, or extranodal deposit) within 1 mm of the mesorectal fascia on MRI. An involved CRM was defined pathologically (post-operative) as a tumour within 1 mm of the CRM. Local recurrence was defined as radiological/endoscopic evidence of disease recurrence in the true pelvis after previous curative excision and was verified pathologically where possible. Time to local or systemic recurrence was the interval from the date of surgery to the first date of diagnosis of recurrence (radiological or pathological). Overall survival was measured from the date of surgery to the recorded date of death from rectal cancer, with the cause of death ascertained from the death certificate where possible.

Statistical analysis: The Chi-squared test was used to compare categorical variables. The t-test was used for normally distributed continuous data. Statistical analysis was done using software available at http://www.brightstat.com [16].

## Results

**Figure 1:**
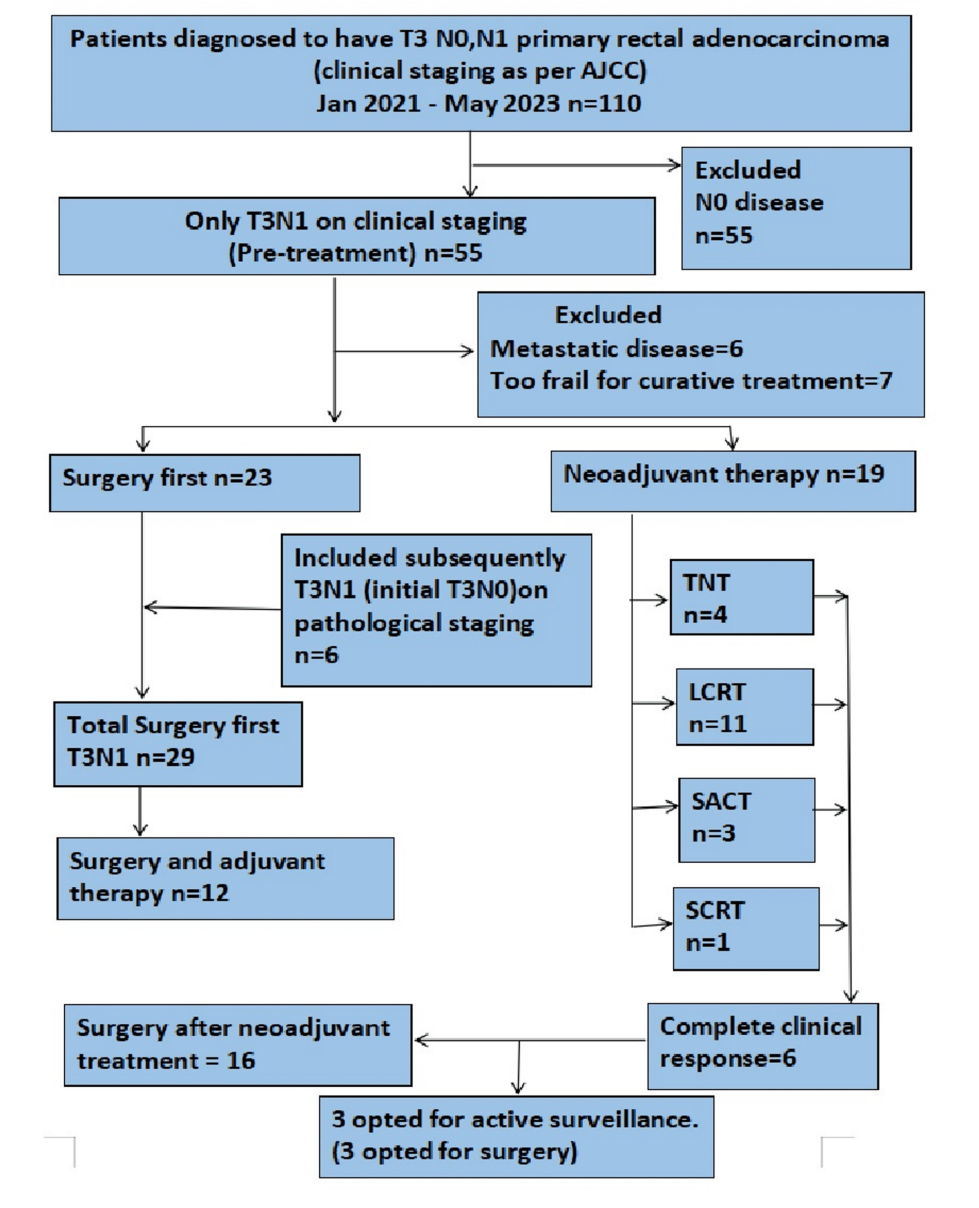
STROBE diagram representing patient cohort Schematic representation of the number of patients included and excluded. STROBE-STrengthening the Reporting of OBservational studies in Epidemiology; AJCC-American Joint Cancer Committee; TNT-Total Neoadjuvant Therapy; LCRT-Long course chemoradiotherapy; SACT-Systemic anticancer therapy; SCRT-Short course radiotherapy; n-Number of patients.

One hundred and ten patients with suspected borderline stage II/stage III (clinical/radiological T3N0 and T3N1) rectal adenocarcinoma were initially identified in the period between January 2021 and May 2023. Patients with T3N1 disease alone were initially included in the study. Six additional patients who were T3N0 on clinical staging and who on pathological staging turned out to be T3N1 were also included in the study. Six patients were found to have metastatic disease, and seven patients were too frail to be appropriate for curative treatment; hence, both groups were excluded. Forty-eight patients were finally included in the study. Twenty-nine patients underwent surgery first, and 19 patients were advised neo-adjuvant therapy. Of the 19 patients who underwent neoadjuvant treatment, 11 had LCRT, four received TNT (SCRT and four cycles of CAPOX), three received SACT (RT was omitted due to prior RT), and only one received SCRT (Figure 1).

No significant difference was noted in age, sex distribution, performance status, or American Society of Anaesthesiologists (ASA) grading between the two groups (Table 1). A significantly higher portion of patients in the surgery-first group had mid-rectal cancers, while the neoadjuvant group had predominantly low rectal cancers. Nine patients (47.3%) in the neoadjuvant group had a threatened CRM while 12 patients (63.2%) had extramural vascular invasion (EMVI +ve). Only one patient had a threatened CRM (3.4%) in the surgery first group, while nine were EMVI +ve (31%). This did result in a statistically significant difference between the two groups (Table 1).

**Table 1:** Demographics and peri-operative clinical data CRM-Circumferential resection margin; EMVI-Extramural vascular invasion; PS-Performance status; ASA-American Society of Anaesthesiologists; APER-Abdominoperenial excision of rectum; CCR-Complete clinical response. *All p-value calculations were done using the Chi-square test (unless otherwise specified); p-value <0.05 considered significant; ^Calculated using the Student t-test. Data is represented as the number of patients along with percentages in parenthesis unless otherwise specified.

	Surgery first (n=29)	Neoadjuvant group (n=19)	p-value*
Median Age in years (Range)	70 (53-85)	68 (58-79)	0.739^
Sex Ratio (Male:Female)	19:10	10:09	0.395
Tumour location			
Upper rectum	7 (24.1%)	1 (5.3%)	0.0019
Mid rectum	14 (48.3%)	4 (21%)	0.0336
Lower rectum	8 (27.6%)	14 (73.7%)	0.0017
Risk stratification			
Pre-Op CRM threatened	1 (3.4%)	9 (47.3%)	0.0007
EMVI +ve	9 (31%)	12 (63.2%)	0.018
PS (Median)	1	0	
ASA (Median)	2	2	
Procedure			<0.001
Anterior Resection	25 (86.2%)	4 (21%)	
APER	2 (6.9%)	12 (63.2%)	
Surveillance (CCR)	0	3 (15.8%)	
Panproctocolectomy	2 (6.9%)	0	

A larger proportion of patients underwent an abdominoperenial excision of the rectum (APER) in the neoadjuvant group as they had relatively lower-positioned cancers relative to the surgery first group. Two patients in the surgery-first group underwent panproctocolectomy. One patient had a synchronous dysplastic caecal polyp with a family history of ulcerative colitis. The other patient had rectal cancer on a background of active ulcerative colitis, necessitating more extensive surgery.

Among patients who had a complete clinical response, three (15.8%) underwent surgery and three (15.8%) attempted a surveillance strategy. On histopathology examination, the patients that were found to have T2 or less were five (17.24%) and seven (43.75%) in the surgery-first and neo-adjuvant therapy groups, respectively. The total for the neoadjuvant group here is taken as 16 as three patients who had a complete clinical response to neo-adjuvant treatment did not undergo surgery]. In both groups, one patient was found to have T4 disease on histopathology (3.4% vs 6.3%) (Table 2).

**Table 2:** Histopathological outcomes *Six cases had a complete clinical response, three were on surveillance, and three were taken for surgery. A p-value <0.05 was considered significant; *p-value calculation using the Kruskal-Wallis Test; ^p-value calculation using Chi-square test unless otherwise specified; Data represented as the number of patients with percentages in parenthesis, unless otherwise specified.

	Surgery first group	Neoadjuvant group	p value^
Tumour			0.032*
ypT0		3* (15.7%)	
pT1/ypT1	3 (10.3%)	1 (5.2%)	
pT2/ypT2	2 (6.9%)	3 (%)	
pT3/ypT3	23 (79.3%)	8 (50%)	
T3a	1	1	
T3b	12	5	
T3c	9	2	
T3d	1	0	
pT4/ypT4	1 (3.4%)	1 (6.3%)	
Node			0.18*
pN0/ypN0	12 (41.4%)	10 (62.5%)	
pN1/ypN1	16 (55.2%)	6 (37.5%)	
pN2/ypN2	1 (3.4%)		
CRM involvement	0	1 (6.25%)	0.47
EMVI positive	12 (41.4%)	3 (4.8%)	0.12
EMLI positive	13 (44.8%)	3 (4.8%)	0.08
Perineural invasion	6 (20.7%)	3 (4.8%)	0.704
Median CRM distance from tumour in mm	10	10	0.272
Intra-operative perforation	0	1 (6.25%)	0.47
Nodal positivity	13 (44.8%)	3 (4.8%)	0.0352
Degree of differentiation			0.557
Moderate	28 (96.5%)	16 (100%)	
Poor	1 (3.5%)	0	
pT3N1	13 (44.8%)	4 (25%)	

On pathological staging, 12 (41.4%), 13 (44.8%), and six (20.7%) patients were found to have EMVI, extramucosal lymphatic invasion (EMLI), and perineural invasion in the surgical group. EMVI was higher than what was estimated on clinical staging (nine (31%) vs 12 (41.4%)). In the neoadjuvant group, the EMVI, EMLI, and perineural invasions were all three (18.75%), showing a good response to pre-operative treatment (Table 2). Only seven of the 23 (30.43%) cases that were deemed to be N1 on pretreatment staging were found to have node-positive disease. Similarly, six of the 55 (10.9%) cases, thought to be T3N0, showed node-positive disease. There was no significant difference in the T3 substaging among both groups, indicating a similar risk profile.

Local recurrence was not noted in any case during the follow-up period in either group. The median follow-up period among the surgery first group was 30 months and in the neoadjuvant group was 26 months.

**Figure 2:**
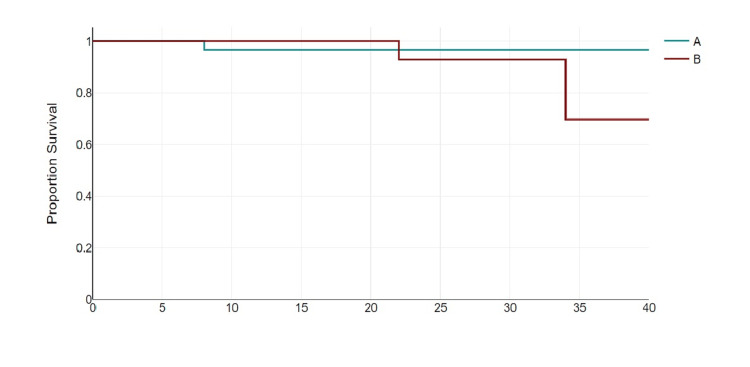
Kaplan-Meier plot showing overall survival in both groups A-Surgery first group, B-Neoadjuvant group. Chi-square=1.19, p=0.275(Log-rank test).

Two cases in the surgery first group and one case in the neoadjuvant group had distant spread (no statistical significance was noted, p=1.0). Only one death was noted in the surgery-first group vs two in the neoadjuvant group (Table 3). One of the deaths in the neoadjuvant group had concomitant oesophageal cancer, and it was difficult to determine which cancer was responsible for the metastasis (in this case, the spread was more likely to be due to oesophageal cancer). The median disease-free survival (DFS) in the surgery first group was longer (29.5 vs 22 months) and found to not be statistically significant. This difference may have been exacerbated by the above-mentioned patient. A Kaplan-Meier plot showing overall survival in both groups is shown in Figure 2.

**Table 3:** Clinical outcomes *One death attributable to concomitant oesophageal cancer diagnosed earlier; p-value calculation using chi-square test; p<0.05 was considered significant; ^Log-rank test Data is represented as the number of patients with percentages in parenthesis unless otherwise specified.

		Surgery first group	Neoadjuvant group	p-value
CT	Local Recurrence	0	0	
	Distant recurrence	2(6.9%)	1(5.26%)	1.0
Colonoscopy	Local Recurrence	0	0	
Combined	Local Recurrence	0	0	
	Distant recurrence	2(6.9%)	1(5.26%)	1.0
Deaths		1	2*	0.569
Median disease-free survival (DFS)- months		29.5	22	<0.275^
Median overall survival (OS)- months		30	22	<0.263^

## Discussion

Our results demonstrate the non-inferiority of a surgery-first approach in T3N1 rectal cancers. We have demonstrated the striking discrepancy between the preclinical assessment of nodal disease with that of nodal cancer deposits on histopathology, potentially altering the stage and choice of initial treatment. This implies we may be offering chemotherapy in cases where it may not be necessary and can avoid potential toxicities of chemotherapy. What is clear from the study is that CRM positivity will require some form of neoadjuvant therapy. EMVI positivity doesn’t preclude a surgery-first approach as adjuvant therapy can always be given.

The crux of the issue is pre-treatment MRI staging. Very often, reactive nodes are as large, if not larger, than those with nodal metastasis. Border contour and signal intensity characteristics on MRI improve the diagnostic accuracy for a more comprehensive staging and potentially reduce exposure to unnecessary radiotherapy (RT) as accurate pre-operative staging is important in classifying the nodal burden in this cohort. The study by Brown et al. clearly demonstrated the challenges as well as the importance of MRI determination of nodal disease as the staging process and subsequent treatment rests on this finding [17].

The 2017 ESMO guidelines, used ubiquitously across the UK, recommend a risk-adapted strategy for locally advanced rectal cancer considering tumour position, T3 substage, CRM involvement, and EMVI. For intermediate/locally advanced rectal cancers(cT3a/b) with a favourable risk profile, i.e., no EMVI and a clear mesorectal fascia (MRF), the routine administration of preoperative RT for the nodal disease has been deemed controversial [12]. This is precisely the question we have attempted to answer.

Treatment modalities do vary globally. The National Comprehensive Cancer Network (NCCN) recommends either neoadjuvant/definite immunotherapy or TNT in select cases, followed by surgery for T3N1M0 rectal cancers [18]. The recently conducted PROSPECT trial (Canada, USA, and Switzerland) for locally advanced rectal cancer compared the effects of neoadjuvant folinic acid, fluorouracil, and oxaliplatin (FOLFOX) with the standard chemoradiotherapy for T2-node positive, T3-node negative, and T3-node positive disease among patients who were candidates for sphincter sparing surgery. It found neoadjuvant FOLFOX to be non-inferior to CRT with respect to disease-free survival [19]. An analysis of patient-reported outcomes from the trial revealed lower rates of fatigue and better sexual function 12 months after surgery among patients randomized to the chemotherapy arm who did not receive radiotherapy [20]. This further strengthens the body of evidence in avoiding radiotherapy where there is no clear advantage in either local recurrence or survival rates.

The 2022 Stellar trial compared TNT (SCRT followed by four cycles of CAPOX) to standard CRT among T3/T4 node-positive cases and found the former to be non-inferior with respect to loco-regional recurrence and improved overall survival at three years, albeit with a higher but acceptable level of toxicity [13]. Our study did implement this regimen in four patients but couldn’t replicate similar results as the cohort was too small to be of significance.

In the case of a threatened CRM, it is clear that neo-adjuvant therapy results in a significant reduction (p=0.027) between clinical and pathological staging. All cases in our study had an R0 resection. Whether this translated into a survival benefit or a reduction in local recurrence rates alone is difficult to ascertain in this study as there was no significant difference between the two groups with respect to the CRM (on pathological staging/recurrence rates). A large Finnish study, though, has shown that a threatened CRM (<1 mm) was a risk factor for local recurrence but was not associated with poorer survival [21]. Interestingly enough, all three cases that were put on surveillance post-CCR had a threatened CRM, and none in the follow-up period (26-31 months) had local/systemic recurrence.

There was a significant difference in pre-operative EMVI rates relative to those noted on pathological staging in both groups. This was attributable to an increase in the number of EMVI-positive cases detected in the surgery-first group as well as an expected decrease in the neo-adjuvant group. This correlates with the literature evidence of preoperative EMVI detection in MRI having a sensitivity ranging from 28.2-62% [21,22]. Newer modalities like radiomics models might prove superior in the future to conventional dynamic contrast-enhanced magnetic resonance imaging (DCE-MRI) for EMVI identification [23]. The aforementioned Finnish study predicted a poorer survival benefit with EMVI positivity (on MRI detected preoperatively) [21]. Our results seem to corroborate these findings. All three cases of distant recurrence in our study had pathological EMVI positivity (two in the surgical group and one in the neoadjuvant group). All three deaths noted in our study had pathological EMVI positivity. Interestingly, in one of these cases, EMVI was not detected pre-operatively. This seems to suggest that EMVI positivity is an excellent prognostic factor, and there is a high risk of micrometastasis early on in the disease, which had already occurred at the time of the MRI [22]. Despite having a significant reduction in EMVI positivity (with neoadjuvant therapy), that did not translate into a survival benefit nor a reduction in recurrence rates as per our study. We do accept that a longer follow-up period may alter these results, and hence, the study is still ongoing. A recent Chinese study has demonstrated that those cancers that become EMVI-negative post-neoadjuvant chemoradiotherapy (nCRT) have a better prognosis with improved overall survival rates [24]. This, though, doesn’t preclude a surgery-first approach as chemotherapy can always be given as adjuvant therapy if EMVI positivity is noticed. EMVI positivity doesn’t seem to negatively affect the outcomes of a surgery-first approach, rather it serves as a prognostic tool to determine the need for chemotherapy. Twelve out of 29 cases taken up for a surgery-first approach were given adjuvant chemotherapy in our study.

Interestingly, 12 cases of radiologically detected node-positive disease in the surgery first group had no positive nodes on pathological staging. Seven cases where nodes were not appreciated pre-operatively had positive nodes on histopathology, reiterating the fact that MRI staging of nodal disease is crucial and, at the same time, challenging [19]. This is extremely significant as it fundamentally alters the staging of the cancer and, hence, the way treatment is planned.

Only one patient in this study had a seemingly complete clinical response and underwent surgery but had node-positive cancer. This does correlate with the 25-33% recurrence risk described in the literature, stressing the importance of intense follow-up and surveillance. Current evidence is in favour of the wait-and-watch approach as salvage rates by surgery are upwards of 84% with the promise of organ preservation and superior quality of life outcomes [25].

The more recent trials, for obvious reasons, have focused on much larger cohorts of patients with relatively more advanced disease and significant adverse features. They have focused on optimal pre-operative treatment modalities without questioning the necessity of pre-op treatment in advanced disease.

We concede that the risk profile in the neo-adjuvant group was higher on clinical staging, particularly with reference to a threatened CRM and EMVI, but on pathological staging that difference turned out to be far less pronounced. We also concede that some of the cases included in our study were initially T3N0 (radiological) but histopathologically turned out to be T3N1 (post-surgery). These were also included, as they had no downstaging treatment. We understand that this inclusion may not be universally acceptable, but this further strengthens our observation that a significant portion of cases was actually T3N1 and responded well with a surgery-first approach. We also accept that due to the specificity of the cohort (T3N1 rectal cancers), we could not achieve a larger sample size. Also, as both groups were not randomized, there may have been some element of selection bias (the neo-adjuvant group had relatively lower positioned tumours and a higher percentage of risk factors, i.e., threatened CRM and EMVI positivity). The disease-free survival difference between the two groups was not significant despite being complicated by one patient who died soon after diagnosis due to complications secondary to an already existing oesophageal cancer.

## Conclusions

Based on our single-site MDT experience, we recommend opting for a surgery-first approach in T3N1 rectal cancers, provided the CRM is not threatened. This would provide the best chance of a complete cure from an oncological point of view and would also be technically easier without prior CRT. Adjuvant therapy can always be offered if necessary. EMVI positivity doesn’t preclude surgery but may obviate adjuvant therapy to reduce the risk of recurrence. It also has proven to be the most important prognostic factor for distant recurrence. Most importantly, we have shown a discordance between radiological staging and the final histopathological staging. Despite this, outcomes have been similar between the surgery-first and neoadjuvant treatment groups. Hence, opting for a surgery-first approach may allow some patients to avoid the side effects of adjuvant therapy. If the patient is averse to surgery and wishes to see if a complete clinical response is possible, then CRT is recommended.

## References

[1] Braun M (2024). T3N1M0 rectal cancer: the optimal initial management is systemic anti-cancer therapy. Br J Surg.

[2] Harris DA (2024). T3 N1 M0 rectal cancer: optimal initial management is upfront surgery. Br J Surg.

[3] Nilsson PJ (2024). T3 N1 M0 rectal cancer: optimal initial management is total neoadjuvant therapy. Br J Surg.

[4] Arthur C, Teo M (2024). T3 N1 M0 rectal cancer: the optimal initial management is long-course chemoradiotherapy. Br J Surg.

[5] Bruheim K, Guren MG, Skovlund E (2010). Late side effects and quality of life after radiotherapy for rectal cancer. Int J Radiat Oncol Biol Phys.

[6] Rothenberger DA, Akbari R, Baxter NN (2004). Are we overtreating some patients with rectal cancer?. Oncology (Williston Park).

[7] Basch E, Dueck AC, Mitchell SA (2023). Patient-reported outcomes during and after treatment for locally advanced rectal cancer in the PROSPECT trial (Alliance N1048). J Clin Oncol.

[8] Pappou EP, Temple LK, Patil S (2022). Quality of life and function after rectal cancer surgery with and without sphincter preservation. Front Oncol.

[9] Wasmuth HH, Færden AE (2024). The Norwegian Watch and Wait study: Norwait for rectal cancer. A report from a failed study-a word of caution. Updates Surg.

[10] Duraes LC, Kalady MF, Liska D (2023). Word of caution: rectal cancer without response to neoadjuvant treatment - do not wait for surgery. Am J Surg.

[11] Fernández E (2005). Observational studies in epidemiology (STROBE) (Article in Spanish). Med Clin (Barc).

[12] Glynne-Jones R, Wyrwicz L, Tiret E, Brown G, Rödel C, Cervantes A, Arnold D (2017). Rectal cancer: ESMO Clinical Practice Guidelines for diagnosis, treatment and follow-up. Ann Oncol.

[13] Jin J, Tang Y, Hu C (2022). Multicenter, randomized, phase III trial of short-term radiotherapy plus chemotherapy versus long-term chemoradiotherapy in locally advanced rectal cancer (STELLAR). J Clin Oncol.

[14] Miles WE (1971). A method of performing abdomino-perineal excision for carcinoma of the rectum and of the terminal portion of the pelvic colon (1908). CA Cancer J Clin.

[15] (2024). Colorectal cancer: NICE clinical guideline. https://www.nice.org.uk/guidance/ng151/resources/colorectal-cancer-pdf-66141835244485.

[16] Stricker D (2008). BrightStat.com: free statistics online. Comput Methods Programs Biomed.

[17] Brown G, Richards CJ, Bourne MW, Newcombe RG, Radcliffe AG, Dallimore NS, Williams GT (2003). Morphologic predictors of lymph node status in rectal cancer with use of high-spatial-resolution MR imaging with histopathologic comparison. Radiology.

[18] Benson AB, Venook AP, Adam M (2024). NCCN Guidelines® insights: rectal cancer, version 3.2024. J Natl Compr Canc Netw.

[19] Schrag D, Shi Q, Weiser MR (2023). Preoperative treatment of locally advanced rectal cancer. N Engl J Med.

[20] Yu X, Song W, Guo D (2020). Preoperative prediction of extramural venous invasion in rectal cancer: comparison of the diagnostic efficacy of radiomics models and quantitative dynamic contrast-enhanced magnetic resonance imaging. Front Oncol.

[21] Sohn B, Lim JS, Kim H, Myoung S, Choi J, Kim NK, Kim MJ (2015). MRI-detected extramural vascular invasion is an independent prognostic factor for synchronous metastasis in patients with rectal cancer. Eur Radiol.

[22] Kim TH, Woo S, Han S, Suh CH, Vargas HA (2019). The diagnostic performance of MRI for detection of extramural venous invasion in colorectal cancer: a systematic review and meta-analysis of the literature. AJR Am J Roentgenol.

[23] Siddiqui MR, Simillis C, Hunter C (2017). A meta-analysis comparing the risk of metastases in patients with rectal cancer and MRI-detected extramural vascular invasion (mrEMVI) vs mrEMVI-negative cases. Br J Cancer.

[24] Chen M, Ma Y, Song YW, Huang J, Gao YH, Zheng J, He F (2023). Survival outcomes of different neoadjuvant treatment regimens in patients with locally advanced rectal cancer and MRI-detected extramural venous invasion. Cancer Med.

[25] Bahadoer RR, Peeters KC, Beets GL (2021). Watch and wait after a clinical complete response in rectal cancer patients younger than 50 years. Br J Surg.

